# The Timing and Magnitude of the Type I Interferon Response Are Correlated with Disease Tolerance in Arbovirus Infection

**DOI:** 10.1128/mbio.00101-23

**Published:** 2023-04-25

**Authors:** Alexandra Hardy, Siddharth Bakshi, Wilhelm Furnon, Oscar MacLean, Quan Gu, Margus Varjak, Mariana Varela, Muhamad Afiq Aziz, Andrew E. Shaw, Rute Maria Pinto, Natalia Cameron Ruiz, Catrina Mullan, Aislynn E. Taggart, Ana Da Silva Filipe, Richard E. Randall, Sam J. Wilson, Meredith E. Stewart, Massimo Palmarini

**Affiliations:** a MRC-University of Glasgow Centre for Virus Research, Glasgow, Scotland, United Kingdom; b School of Biology, Centre for Biomolecular Sciences, University of St. Andrews, St. Andrews, Fife, United Kingdom; Indiana University Bloomington

**Keywords:** arbovirus, disease tolerance, innate immunity, interferons

## Abstract

Infected hosts possess two alternative strategies to protect themselves against the negative impact of virus infections: resistance, used to abrogate virus replication, and disease tolerance, used to avoid tissue damage without controlling viral burden. The principles governing pathogen resistance are well understood, while less is known about those involved in disease tolerance. Here, we studied bluetongue virus (BTV), the cause of bluetongue disease of ruminants, as a model system to investigate the mechanisms of virus-host interactions correlating with disease tolerance. BTV induces clinical disease mainly in sheep, while cattle are considered reservoirs of infection, rarely exhibiting clinical symptoms despite sustained viremia. Using primary cells from multiple donors, we show that BTV consistently reaches higher titers in ovine cells than cells from cattle. The variable replication kinetics of BTV in sheep and cow cells were mostly abolished by abrogating the cell type I interferon (IFN) response. We identified restriction factors blocking BTV replication, but both the sheep and cow orthologues of these antiviral genes possess anti-BTV properties. Importantly, we demonstrate that BTV induces a faster host cell protein synthesis shutoff in primary sheep cells than cow cells, which results in an earlier downregulation of antiviral proteins. Moreover, by using RNA sequencing (RNA-seq), we also show a more pronounced expression of interferon-stimulated genes (ISGs) in BTV-infected cow cells than sheep cells. Our data provide a new perspective on how the type I IFN response in reservoir species can have overall positive effects on both virus and host evolution.

## INTRODUCTION

Many pathogens infect more than one animal species, resulting in dramatically different clinical outcomes as a result of complex differences in pathogen-host interactions (1–3). The infected host can protect itself from diseases using essentially two distinct strategies: resistance and tolerance (4–7). Resistance refers to the host immune response’s ability to hamper and eliminate the pathogen once infection has occurred. Conversely, tolerance (also referred to as resilience and not to be confused with immunological tolerance) is a host defense strategy that protects against the deleterious effects of infection and the host immune response in the face of high pathogen burden (8–11). Disease tolerance was first described in the context of the selective pressures exerted by parasites and herbivores on plant evolution. Only relatively recently has this concept been explored in the context of pathogen–mammalian-host interactions (10, 12, 13). A variety of factors, including genetic traits, innate and adaptive immunity, microbiome, and age, have been shown to play a role in disease tolerance (14).

The distinction between resistance and tolerance is of paramount importance in understanding the ecology and epidemiology of infectious diseases. Natural reservoir species of given pathogens can use disease tolerance as an effective mechanism to cope with infection. At the same time, high pathogen burden and extended infection times can favor onward transmission to target susceptible species. For example, some viral infections (Nipah virus and Hendra virus) in different species of bats maintain high viral burdens without apparent disease, while infection with these viruses results in severe disease in humans (15). African buffaloes are tolerant to foot-and-mouth disease virus relative to domestic cattle, while wild hogs are in general tolerant to African swine fever virus as opposed to domestic pigs (16, 17).

In this study, we aimed to dissect virus-host interactions in susceptible and disease-tolerant host species using a tractable experimental system. We used bluetongue virus (BTV), an arbovirus of ruminants with a significant impact on the agriculture sector (18–21). BTV is a double-stranded RNA (dsRNA) virus in the family *Reoviridae* existing in nature as more than 27 serotypes (22–24). BTV can essentially infect all domestic and wild ruminant species, but the clinical manifestations of infection can vary considerably, both between species and between individuals (25, 26). BTV infects and induces viremia in both cattle and sheep, although in general only the latter display the severe clinical signs associated with bluetongue. Therefore, sheep are recognized as susceptible hosts, while cattle are mostly asymptomatic (27–31). Clinical manifestations particularly evident in sheep include fever, nasal discharge, diffuse edema affecting the head and lungs, and hemorrhagic lesions (32–35). In the infected organs, endothelial cells (EC) have been described as major cellular targets of BTV, where virus replication may cause cell injury (36–39). Damage to the EC is believed to be crucial in the pathogenesis of the disease. Indeed, injuries of the EC lining the small blood vessels clearly reflect the clinical signs observed during BTV infection. The increased vascular permeability associated with EC damage may account for the observed edema as well as the reported lesions, such as vascular thrombosis and hemorrhages (36, 37, 40).

Disease in cattle is quite rare, with only intermittent reports of field cases of bluetongue disease in cattle (28, 41). In addition, clinical disease has been very difficult to reproduce experimentally in cattle, regardless of animal age, virus serotype, or route of inoculation (33, 42–47). A possible exception is the North European BTV-8 from 2006, which induced one of the largest outbreaks of bluetongue in history, with elevated mortality in sheep but also some morbidity in cattle (although of reduced severity compared to that in sheep) (42).

In this study, we aimed to investigate whether susceptibility or tolerance to BTV could be correlated with fundamental differences in virus-host cell interactions that can be assessed in *in vitro* experiments. We focused our studies on primary cells derived from sheep and cattle, two animal species that exhibit different clinical outcomes of BTV infection. Our findings provide a unique comparative approach to understand how virus-host interactions may influence disease tolerance and susceptibility to virus infections.

## RESULTS

### Replication kinetics of BTV in bovine and ovine primary cells.

We first investigated whether host resilience or susceptibility to BTV infection could be correlated with the virus replication kinetics in primary cells collected from sheep (susceptible species) or cattle (resilient species). We carried out virus replication assays in primary skin fibroblasts and endothelial cells. We chose primary cells because they are likely to possess an intact cell autonomous innate immune response, unlike many established immortalized cell lines. BTV is an arbovirus that gains access to animals through the skin, and skin fibroblasts were a convenient system to use, although we have no evidence that they are infected *in vivo*. Endothelial cells are instead known targets of BTV infection *in vivo* (36–39).

**FIG 1:**
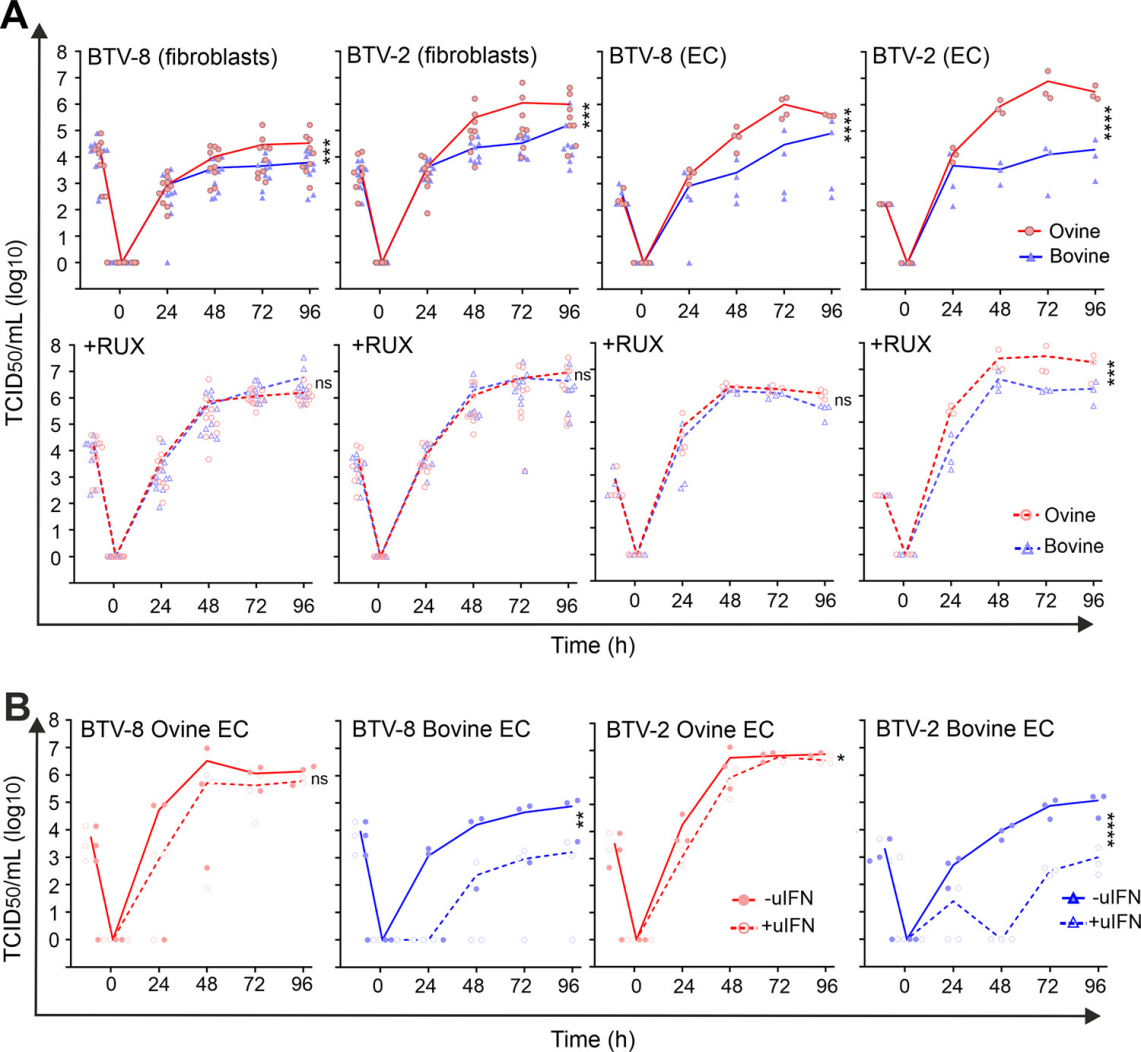
Differences in the replication kinetics of BTV in primary cells are dependent on the type I IFN response. (A) (Top) Virus replication curves of BTV-8 and BTV-2 in ovine (red) and bovine (blue) primary fibroblasts and EC. Data points for independent experiments are shown (for BTV-8, *n* = 10, and for BTV-2, *n* = 8, for ovine and bovine fibroblasts; for BTV-8, *n* = 4, and for BTV-2, *n* = 3, for ovine and bovine EC). (Bottom) The same replication curves in the presence of 4 μM Rux. Experiments in the presence and absence of Rux were carried out at the same time. (B) Virus replication curves of BTV-8 and BTV-2 in ovine (red) and bovine (blue) EC. Cells were mock treated (solid lines) or pretreated with 500 U of uIFN before infection. Independent experiments were repeated for BTV-8 (*n* = 3) and BTV-2 (*n* = 3) for ovine and bovine endothelial cells. For all experiments, cells were infected at an MOI of 0.01, and supernatants were harvested at the indicated time postinfection. Cell-free virus was titrated by endpoint dilution on BSR cells. Values are averages from independent experiments, as indicated, using at least three batches of primary cells independently generated. A one-way ANOVA with the Holm-Šídák *post hoc* correction test was carried out on the values for area under the curve (AUC) to assess statistical significance between conditions. ns, not significant (*P* > 0.05); *, *P* < 0.05; **, *P* < 0.01; ***, *P* < 0.001; ****, *P* < 0.0001.

We were particularly careful in performing these assays in distinct cell preparations collected from multiple donors to compensate for individual and batch-to-batch variation. In addition, we used two distinct BTV serotypes, BTV-8 and BTV-2, to ensure that differences observed were not associated with a single virus strain. In replication assays, both BTV-8 and BTV-2 reached statistically significantly higher titers in ovine fibroblasts (OvFib) and ovine EC (OvEC) than in the corresponding bovine cells (BovFib and BovEC) (Fig. 1A). Interestingly, both BTV-2 and BTV-8 reached titers at times 100-fold higher in OvEC than in BovEC, while differences between titers in ovine and bovine fibroblasts were more modest (about 10-fold). The differences we observed in the virus replication kinetics were correlated with differences in the progression of cytopathic effect (CPE) during the time course of infection (see Fig. S1 in the supplemental material). The monolayer of OvEC was destroyed almost completely by BTV within 72 h postinfection, while BovEC showed restricted foci of infection that did not progress as extensively, even at 96 hpi.

10.1128/mbio.00101-23.1 FIG S1 Cytopathic effect induced by BTV-8 in ovine and bovine primary aorta endothelial cells. Cells were infected with BTV-8 at an MOI of ~0.01, and monolayers were fixed at different times postinfection. Monolayer integrity was assessed by staining cells with Coomassie brilliant blue. Download FIG S1, TIF file, 3.1 MB. Copyright © 2023 Hardy et al. 2023 Hardy et al. https://creativecommons.org/licenses/by/4.0/ This content is distributed under the terms of the Creative Commons Attribution 4.0 International license.

### Differences in the replication kinetics of BTV in bovine and sheep cells are modulated by the IFN response.

BTV is a strong inducer of type I interferon (IFN), both *in vivo* and *in vitro* (48–53). Hence, we investigated whether the antiviral IFN response played a role in the different phenotype displayed by BTV in sheep and cow cells. To this end, we examined virus replication in the presence of ruxolitinib (Rux), an efficient inhibitor of the IFN signaling pathway acting on Janus kinase 1 (JAK1) and JAK2 (54, 55). Higher BTV titers were observed in cells treated with Rux (Fig. 1A, bottom) than in the untreated cells at all time points and in all cell types used. Strikingly, the differences in the BTV titers after Rux treatment were more substantial in bovine cells. Indeed, the addition of Rux was able to rescue BTV replication in bovine cells to levels comparable to those in Rux-treated ovine cells. Hence, the JAK/STAT (signal transducer and activator of transcription) pathway and the initiation of the signaling cascade leading to the regulation of hundreds of IFN-stimulated genes (ISGs) appears to play a role in the different replication kinetics shown by BTV in ovine and bovine cells.

We next performed virus replication assays in cells with or without pretreatment with type I IFN (500 IU/mL universal IFN [uIFN]). The induction of an antiviral state restricted BTV-8 replication, with the effect being more prominent in cow than sheep fibroblasts (Fig. 1B). As EC showed the largest differences in the BTV replication kinetics between sheep and cow cells, we concentrated further on examining these differences. The presence of IFN minimally affected BTV-8 or BTV-2 replication in OvEC, as titers were ~15- to 60-fold lower at 24 hpi in cells pretreated with uIFN than untreated cells, but there were no major differences at later time points for both serotypes tested. Conversely, replication of both BTV-8 and BTV-2 was significantly hampered in BovEC pretreated with uIFN than in untreated cells. Replication of both BTV-8 and BTV-2 was severely delayed, reaching very low titers at early time points, and started to increase only from 48 hpi onward. In BovEC, BTV-2 and BTV-8 reached titers ~40- to 120-fold higher in mock-treated cells than IFN-treated cells (Fig. 1B).

We found that induction of antiviral cytokines in the supernatant of BTV-infected BovFib and BovEC was higher than in sheep primary cells (Fig. S2A). Interestingly, we also found a trend for bovine primary cells to display a stronger antiviral cytokine response than sheep cells when we used, instead of BTV, a preparation of Sendai virus rich in copyback-defective interfering genomes (known activators of the interferon response) (56, 57) (Fig. S2B). However, we found no intrinsic differences in how cow and sheep cells respond to type I IFN, as assessed by either upregulation of Mx1 (Fig. S2C) or induced protection against a vesicular stomatitis virus (VSV) reporter system (Fig. S2D) in response to uIFN.

10.1128/mbio.00101-23.2 FIG S2 Antiviral responses of ovine and bovine primary cells. Box plots showing concentrations of antiviral cytokines released in the supernatants of primary ovine (red) and bovine (blue) cells triggered by BTV-8 (A) or SeV (B) infection for 24 h. Results are expressed in international units per milliliter and were obtained by treating indicator cells for 24 h with serial dilutions of UV-inactivated supernatants (or uIFN as a control) before challenge with VSV-ΔG-GFP for 6 h. The percentage of virus-infected cells (cells expressing GFP gated on single live cells) was measured for each condition. Levels of antiviral cytokines were calculated by fitting a linear regression to a standard curve obtained by diluting known amounts of uIFN. Independent batches of primary cells are shown. Statistical differences between each group were assessed using a Student *t* test with Welch’s correction. (C) Primary cells were stimulated with serial dilutions of uIFN (from 250 to 4 IU/mL) for 24 h or mock treated, and upregulation of Mx1 expression was measured by Western blotting. Detection of α-tubulin was used as a loading control. (D) The protective effect of uIFN treatment was assessed in ovine (red) and bovine (blue) primary fibroblasts (left) and EC (right). Cells were treated with the indicated doses of uIFN for 24 h or left untreated and then challenged with a fixed input of VSV-ΔG-GFP for 6 h. The percentage of infected cells (GFP^+^ cells gated on single live cells) was measured for each condition and normalized to values obtained without uIFN treatment. Download FIG S2, TIF file, 2.3 MB. Copyright © 2023 Hardy et al. 2023 Hardy et al. https://creativecommons.org/licenses/by/4.0/ This content is distributed under the terms of the Creative Commons Attribution 4.0 International license.

Overall, the data obtained so far indicate that the nature of the antiviral response may determine whether a host is susceptible or tolerant to BTV-induced disease.

### Identification of ISGs restricting BTV replication.

**FIG 2:**
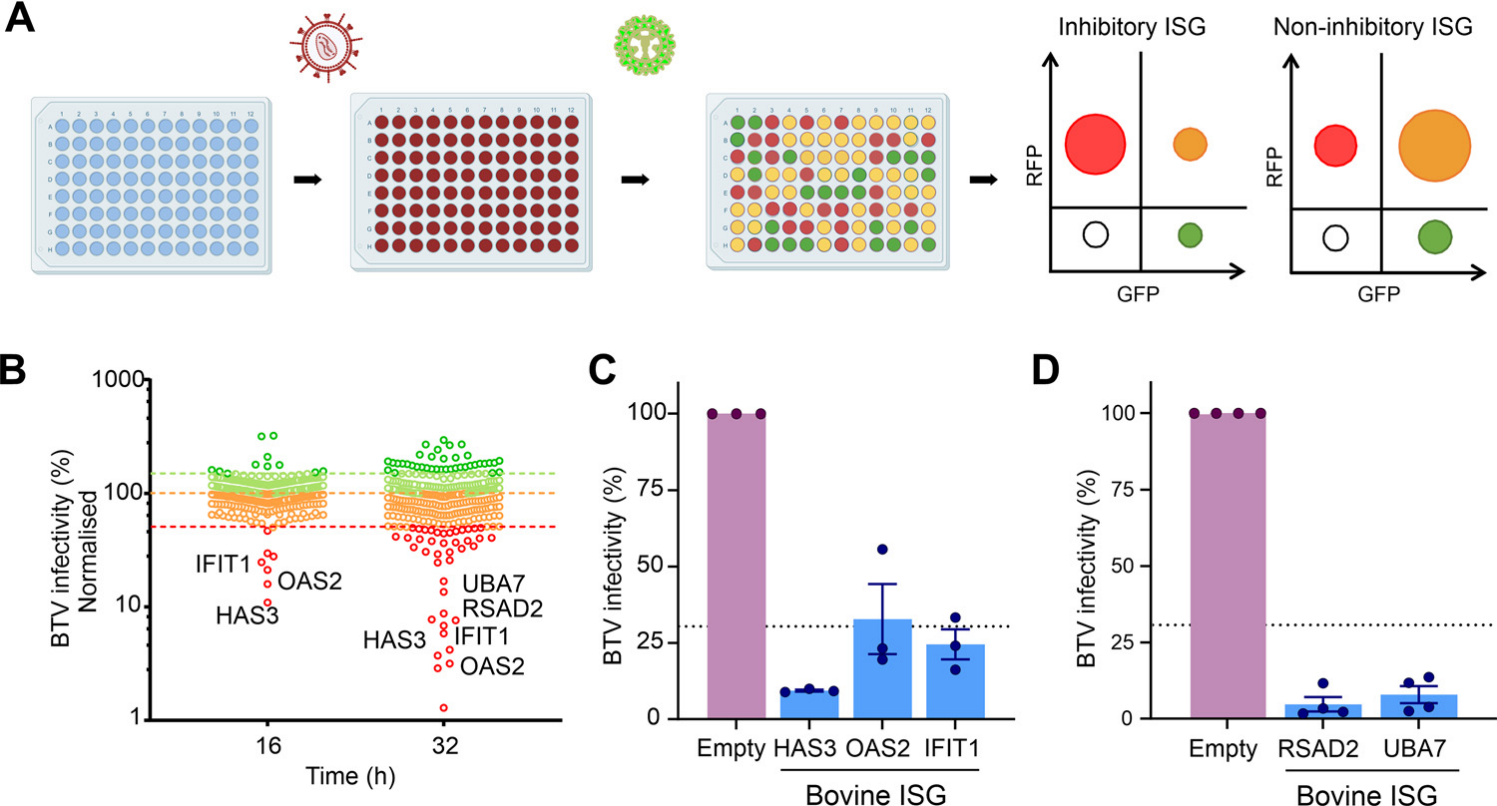
A bovine ISG screen reveals gene candidates with anti-BTV properties. (A) Schematic representation of the bovine ISG overexpression screen. 293T cells were transduced with lentiviruses expressing ISGs and RFP. Cells were subsequently infected with BTV-mGFP. At either 16 or 32 hpi, cells were fixed, and the number of infected cells was determined via flow cytometry. Plots representing the relative importance of different cell populations gated on their expression of GFP (virus-infected cells) or RFP (efficiently transduced cells) for an inhibitory or a noninhibitory ISG are schematically represented. (B) Scatterplot representing the percentage of BTV-mGFP replication for each gene normalized to the median. The screen was done at 16 and 32 hpi. Genes restricting BTV by more than 30% are considered inhibitory. The screen at 32 hpi was undertaken in the presence of 0.625 μg/mL puromycin. (C) Validation of antiviral bovine ISGs inhibiting BTV replication at 16 hpi. Data are means and standard errors from three independent experiments. (D) Validation of antiviral bovine ISG inhibiting BTV replication at 32 hpi. Data are means and standard errors from four independent experiments. Some of the schematic images were obtained from Biorender.com under a laboratory license.

The IFN response appeared to suppress BTV replication more effectively in bovine cells. Hence, we next investigated whether ISGs that are induced upon IFN treatment in bovine cells are efficient at blocking BTV replication, while their ovine orthologues may be less effective in doing so. To identify bovine ISGs which could negatively impact BTV replication, we used a medium-throughput lentivirus-based overexpression screening platform. This system was previously used to identify antiviral genes acting against a range of viruses (58–61) and relies on lentivirus vectors coexpressing the ISG of interest and red fluorescent protein (RFP). HEK-293 cells were challenged with BTV expressing the green fluorescent protein (GFP), and the level of virus replication in the presence of each individual ISG was assessed via flow cytometry (Fig. 2A and B). For our screening purposes, we engineered a reporter BTV expressing a monomeric GFP fused to the NS1 open reading frame (ORF) (BTV-8-mGFP).

We designed and constructed a bovine ISG library including core ISGs conserved across mammalian evolution and those that are most highly upregulated in response to IFN in bovine primary cells, as identified in one of our previous studies (Table S1) (62). We carried out the screens in HEK-293T cells transduced with the lentivirus library and infected cells were fixed at two different times postinfection (16 and 32 h postinfection [hpi]) and then analyzed by flow cytometry. By establishing an arbitrary cutoff value of 30% for the normalized level of BTV replication, we identified 17 potential hits with anti-BTV-8-mGFP properties: 7 genes in the 16-hpi screen and 15 in the 32-hpi screen (Fig. 2B; Table S1). Six of the 7 genes identified in the 16-hpi screen were also identified as restrictive at 32 hpi. Given our experience with ISG screens against a variety of viruses (58–61), we ruled out from subsequent analyses ISGs that (i) have known signaling properties (IRF2, IFIH1, and DDX58), (ii) are able to induce expression of a reporter gene under the control of an interferon-stimulated response element (ISRE) promoter in the same type of assays (IRF-1, CDADC1, CXCR4, ISG20, and STARD8), or (iii) are known to affect the general cell metabolism (IDO1) (63). We also excluded SLFN12L, as it was identified only at the 16-hpi time point and not confirmed in the 32-hpi screen.

10.1128/mbio.00101-23.6 TABLE S1 Data from the bovine ISG overexpression screens, including gene names (including ENSEMBL ID), the percentage of cells successfully transduced by each gene, and the value of BTV-8 infectivity normalized to the mean value obtained across the entire library. Download Table S1, XLSX file, 0.05 MB. Copyright © 2023 Hardy et al. 2023 Hardy et al. https://creativecommons.org/licenses/by/4.0/ This content is distributed under the terms of the Creative Commons Attribution 4.0 International license.

We validated these results using different lentivirus preparations with the remaining hits: HAS3 (hyaluronan synthase 3), IFIT1 (interferon-induced protein with tetratricopeptide repeats 1), OAS2 (2′,5′-oligoadenylate synthetase 2), RSAD2 (radical *S*-adenosyl methionine domain containing 2), and UBA7 (ubiquitin-like modifier-activating enzyme 7), which confirmed their anti-BTV properties. All these ISGs were confirmed to possess anti-BTV properties in the validation assays (Fig. 2C and D).

### Ablation of IFIT1 and RSAD2 reduces BTV sensitivity to IFN.

**FIG 3:**
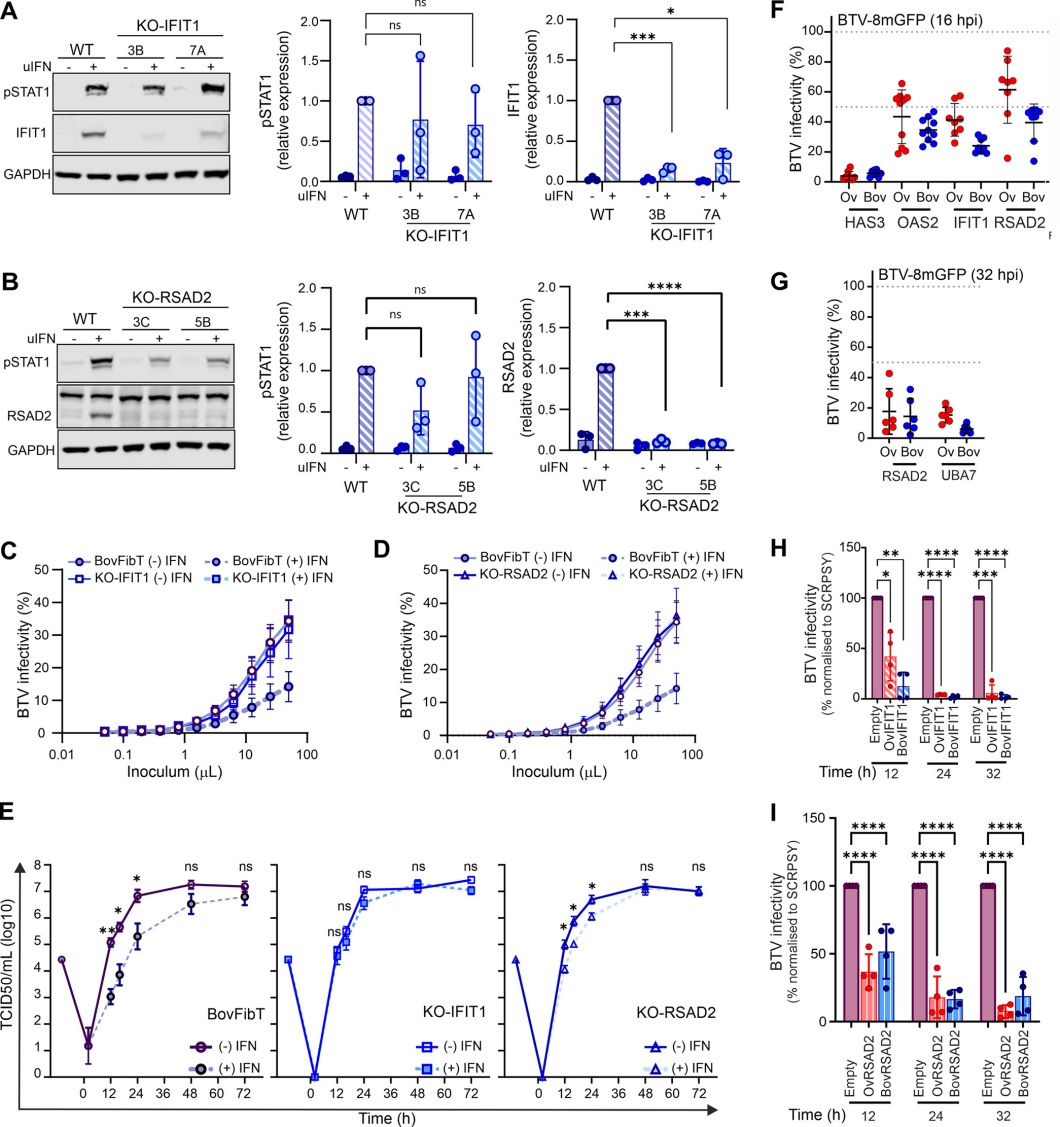
BTV replication is restricted by both cow antiviral ISGs and their sheep orthologues. (A) (Left) Western blot of KO-IFIT1 cells (clones 3B and 7A) and parental BovFibT (WT) showing expression of IFIT1, phosphorylated STAT1, and GAPDH after IFN treatment. (Right) Quantification of Western blots using Image Studio Lite software (LI-COR Biosciences). Data were obtained from 3 independent experiments. (B) (Left) Western blot of KO-RSAD2 cells (clones 3C and 5B) and parental BovFibT (WT) showing expression of RSAD2, phosphorylated STAT1, and GAPDH after IFN treatment. (Right) Quantification of Western blots using Image Studio Lite software (LI-COR Biosciences). Data were obtained from 3 independent experiments. (C) Graph showing infectivity of BTV-8 mGFP in KO-IFIT1 and parental BovFibT in cells pretreated with IFN (1,000 U), or carrier control, before infection with serial dilutions of BTV-8-mGFP for 6 h. Cells were then fixed, and mGFP expression was determined by flow cytometry. (D) Graph showing infectivity of BTV-8 mGFP in KO-RSAD2 and parental BovFibT in cells pretreated with IFN (1,000 U), or carrier control, before infection with serial dilutions of BTV-8-mGFP for 6 h. Cells were then fixed, and mGFP expression was determined by flow cytometry. (E) Virus replication curves of growth of BTV-8 in immortalized BovFibT, KO-IFIT1 cells, and KO-RSAD2 cells in the presence (dashed lines) or absence (solid lines) of 1,000 U uIFN pretreatment. Cells were infected at an MOI of 0.01, and supernatants were harvested at the indicated time points postinfection. Cell-free virus was titrated by endpoint dilution on BSR cells, and values are means and SEM from at least 3 independent experiments. Statistical significance between −uIFN and +uIFN conditions for each time point was calculated using *t* tests with Welch’s correction. (F and G) Relative infectivity of BTV-8-mGFP in 293T cells overexpressing bovine and ovine restriction factors at 16 (F) and 32 (G) hpi (*n* = 8). 293T cells were transduced with lentiviruses expressing the ovine or bovine orthologues for 48 h and infected with BTV-8-mGFP for 16 or 32 h, and mGFP expression was quantified by flow cytometry. BTV infectivity was normalized to the mean obtained from all the negative-control wells. Each dot represents an independent repeat. The mean and standard deviation are presented for each condition. (H and I) Virus titers of BTV-8 in CPT-Tert stably expressing either ovine or bovine IFIT1 (H) and ovine or bovine RSAD2 (I) at the times indicated. Cells stably transfected with an empty lentivirus were used as controls. Cells were infected with BTV-8 (MOI ≈ 0.01), and supernatants were harvested at the indicated time points postinfection. Cell-free virus titers were quantified by endpoint dilution and normalized to titers obtained from the control cell line. Data (*n* =4) are from 2 independent BTV-8 stocks and 2 independently generated stable cell lines for each gene tested. Multiple *t* tests were carried out following a Shapiro-Wilk normality test. ns, not significant (*P* > 0.05); *, *P* < 0.05; ***, *P* < 0.001; ****, *P* < 0.0001.

IFIT1 and RSAD2 are core ISGs conserved across vertebrate evolution and possess broad antiviral activity (62). Furthermore, sheep RSAD2 was shown in a previous study to possess anti-BTV activity (64). To complement the data obtained with our overexpression approach, we used CRISPR/Cas9 to knock out IFIT1 and RSAD2 expression and determine their effect on BTV replication. While we could not successfully generate knockout (KO) cells with the CRISPR/Cas9 system using primary cells, we were successful in immortalized primary bovine fibroblasts (BovFibT). We confirmed the ablation of IFIT1 and RSAD2 by immunoblotting in two clonally selected KO cell populations after stimulation with uIFN for 24 h. The expression of IFIT1 and RSAD2 was either undetectable or greatly reduced in the KO cells in comparison to the parental cells (Fig. 3A and B). Importantly, induction of pSTAT1 in response to uIFN stimulation in KO and parental cells displayed no major differences. These data therefore suggest that the ability of the KO cells to respond to IFN was maintained, although we noticed in the RSAD2-KO cells a somewhat reduced pSTAT1 signal, possibly reflecting clonal variability (Fig. 3B).

We next evaluated the effects of ISGs on the initial stages of viral infection using BTV-8-mGFP. IFIT1-KO cells, RSAD2-KO cells, and parental BovFibT were stimulated with uIFN and then infected with serial dilutions of BTV-8-mGFP for 6 h. As expected, we observed a clear decrease in the number of positive mGFP cells (a proxy for BTV infection) in IFN-treated cells, as opposed to mock-treated cells (Fig. 3C and D). However, we observed no differences in the number of BTV-8-mGFP infected KO cells whether they were pretreated with IFN or mock treated (Fig. 3C and D).

To further assess the effect of IFIT1 and RSAD2 knockout on BTV replication, BovFibT, IFIT-KO cells, and RSAD2-KO cells were either stimulated with uIFN or mock treated for 24 h prior to infection with wild-type BTV-8 (multiplicity of infection [MOI] of 0.01) (Fig. 3E). As expected, pretreatment of BovFibT with IFN suppressed the replication kinetics of BTV-8. On the other hand, pretreatment of IFIT1-KO cells did not affect the replication kinetics of BTV-8. Conversely, IFN stimulation of the RSAD2-KO cells resulted in an intermediate phenotype, with differences in the BTV-8 replication kinetics that were statistically significant compared to untreated cells, but these differences were not as large as those observed in the parental BovFibT. These results provide evidence that the IFN response is significantly less efficient at controlling BTV replication in the absence of IFIT1 and RSAD2.

### No difference in antiviral activity was displayed by sheep and cow ISG orthologues possessing anti-BTV activity.

Our data thus far showed that BTV replication in bovine cells is hampered by the host IFN response. In addition, we also identified at least five bovine ISGs restricting BTV replication. Hence, we next compared the antiviral activity of the ovine and bovine ISG orthologues. We used the exogenous expression assays described above, using independently generated lentiviruses encoding either the ovine or bovine orthologues of IFIT1, RSAD2, OAS2, HAS3, and UBA7. Overall, we did not observe significant differences in the ability of sheep and bovine ISG orthologues to restrict BTV replication at either 16 hpi (Fig. 3F) or 32 hpi (Fig. 3G).

As the above-described experiments were undertaken in a human cell line with a reporter virus, we further validated the results obtained using a sheep cell line (CPT-Tert) stably expressing either IFIT1 or RSAD2. As in the previous experiments, both sheep and bovine IFIT1 and RSAD2 significantly restricted BTV replication (Fig. 3H and I).

### Relative impairment of BTV replication in BovEC is detectable as early as 4 h postinfection.

**FIG 4:**
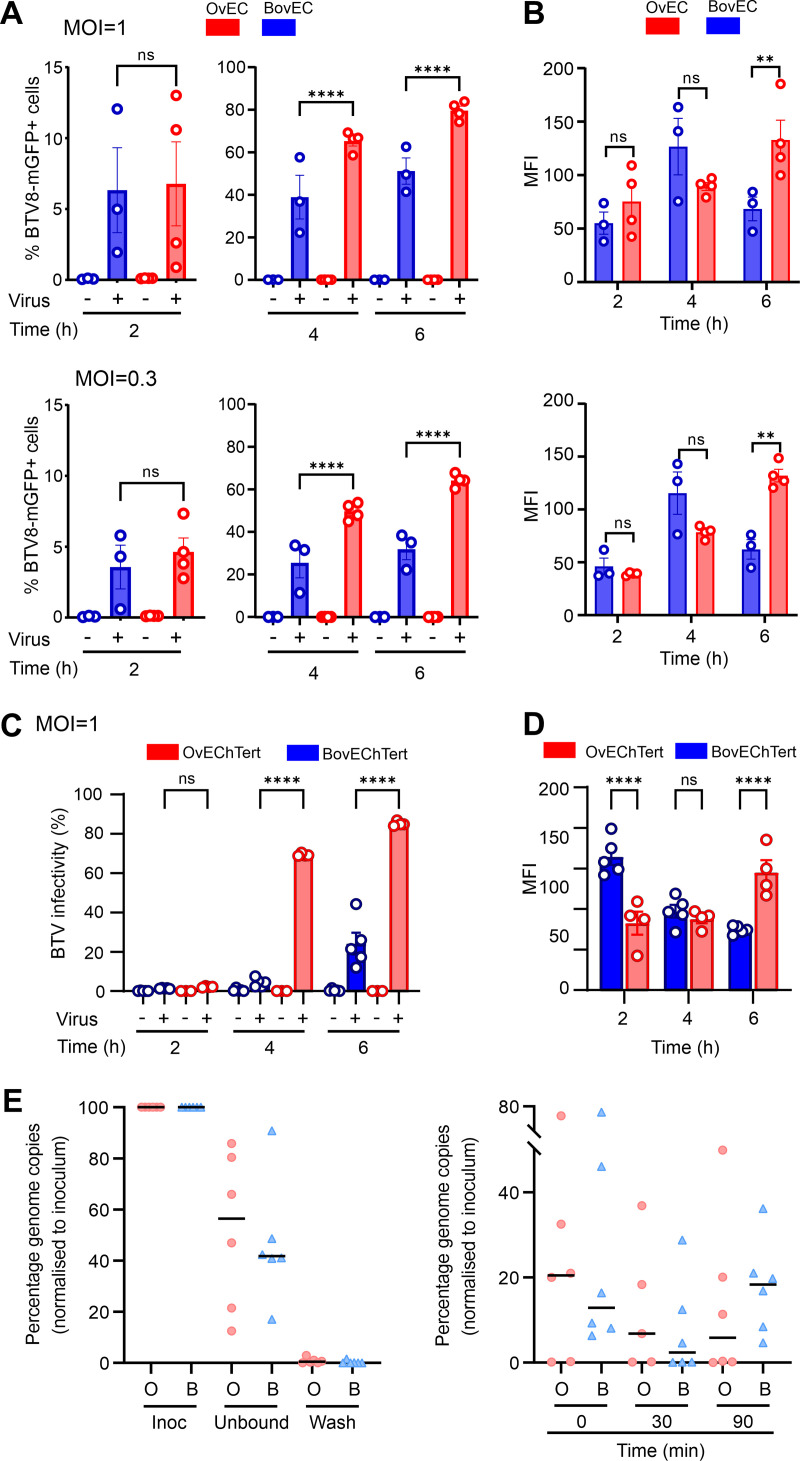
Relative restriction of BTV replication in bovine cells occurs within the first 4 h postinfection. (A) Infectivity of BTV-8-mGFP in primary OvEC and BovEC. Cells were infected with BTV-8-mGFP at an MOI of ~1 (top) or 0.3 (bottom) and fixed at 2, 4, and 6 hpi. mGFP expression was determined by flow cytometry. Data were obtained from 4 independent experiments. (B) Mean fluorescent intensity (MFI) of the infected primary OvEC and BovEC populations at different times postinfection. Data are from 4 independent experiments at two different MOI. (C) Infectivity of BTV-8-mGFP in OvEChTert and BovEChTert cells. Experiments were carried out as for panel A with an MOI of ~1. (D) MFI of the infected OvEChTert and BovEChTert. Data for panels C and D were obtained using the immortalized BovEC from 5 independent experiments using two different clones, and data from immortalized OvEC are from 4 independent experiment using one clone. (E) Binding and entry assays of BTV-8 into OvEChTert and BovEChTert cells assessed by qRT-PCR for BTV segment 10. Cells were synchronously infected with BTV-8 at an MOI of ~10 at 4°C, and samples were taken from the inoculum, unbound virus, or the wash (left) or harvested at 0, 30, and 90 min postinfection (right). Samples were normalized to the inoculum (set at 100%). Data are from 6 independent experiments. ns, not significant (*P* > 0.05); **, *P* < 0.01; ****, *P* < 0.0001.

Next, we further dissected the early events of the BTV life cycle using BTV-8-mGFP to better assess small differences in the early events of BTV replication in BovEC and OvEC. We synchronized infection of BovEC and OvEC and incubated cells for either 2, 4, or 6 hpi before flow cytometry. At 2 hpi, there was no significant difference in the number of infected OvEC and BovEC, but by 4 hpi, there was a significant increase in the number of BTV-mGFP-infected OvEC (Fig. 4A). The mean fluorescent intensities of the two cell types were similar at 2 and 4 hpi, while they became significantly higher in infected OvEC at 6 hpi, suggesting greater translation of BTV proteins in these cells at this time point (Fig. 4B). We repeated the experiments described above in immortalized ovine and bovine EC (OvEChTert and BovEChTert) and obtained similar results (Fig. 4C and D). These data suggest that the differences in the replication kinetics of BTV in OvEC and BovEC can be detected at least as early as 4 hpi.

We next tested whether BTV could enter OvEC more efficiently than BovEC. To this end, we infected immortalized OvEC and BovEC (MOI ≈ 10) by spinoculation and harvested samples from the inoculum, the inoculum after spinoculation for 1 h at 4°C (unbound fraction), and cells at 0, 30, and 90 min postinfection. We then extracted and quantified viral RNA using a quantitative reverse transcription-PCR (qRT-PCR) assay for BTV segment 10 and normalizing values to that for the inoculum. We observed no significant differences on the amount of BTV-8 RNA binding and entering immortalized OvEC compared to BovEC (Fig. 4E). Collectively, the data obtained so far suggest that the relative impairment of BTV replication in BovEC begins as early as 4 hpi.

### BTV-induced protein shutoff is more pronounced in ovine cells and downregulates ISG expression.

**FIG 5:**
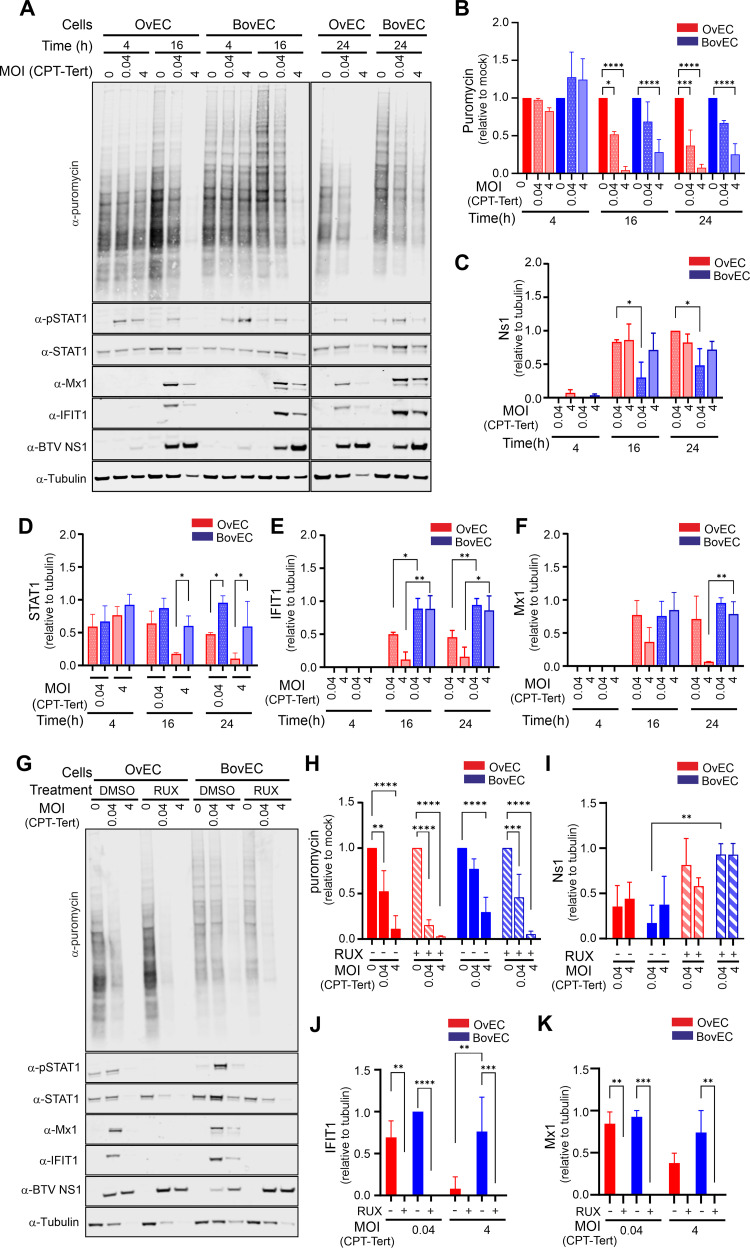
Virus-induced global host cell protein shutoff by BTV is faster and more pronounced in ovine than bovine endothelial cells. (A) Immunoblotting of phosphorylated STAT1, total STAT1, Mx1, IFIT1, and BTV NS1 and protein translation rates (puromycin labeling treatment for 1 h) in BovEC and OvEC infected with BTV-8. Cells were infected at 0.04 or 4 MOI equivalents (measured in the indicator cell line CPT-Tert) and harvested at the time points indicated. (B to F) Relative quantification of immunoblots as in panel A obtained from 3 independent experiments. (B) Quantification of relative protein shutoff over the course of BTV infection as determine by puromycin incorporation. All values were normalized to the mock (set to 1) for each time point. (C) Relative expression of BTV NS1 over time normalized to tubulin in OvEC and BovEC infected with BTV-8. (D) Expression of STAT1 (relative to mock infection) over time normalized to tubulin in OvEC and BovEC infected with BTV-8. (E) Relative expression of IFIT1 over time normalized to tubulin in OvEC and BovEC. Cells were infected with BTV-8 using 2 different MOI and harvested at 3 different time points. Data are from 3 independent experiments. (F) Relative expression of Mx1 over time normalized to tubulin in OvEC and BovEC. Cells were infected with BTV-8 using 2 different MOI and harvested at 3 different time points. Data are from 3 independent experiments. (G) Immunoblotting of phosphorylated STAT1, total STAT1, IFIT1, Mx1, and BTV NS1 and protein translation rates (puromycin labeling treatment for 1 h) in OvEC and BovEC pretreated with 4 μM Rux and infected with BTV-8. Cells were infected at an MOI of 0.04 or 4 and harvested at 24 hpi. (H to K) Relative quantification of immunoblots, as shown in panel G, obtained from 3 independent experiments. (H) Relative protein shutoff in OvEC and BovEC infected with BTV as determined by puromycin incorporation. All values were normalized to the mock-infected control. (I) Relative expression of BTV NS1 (relative to tubulin) in OvEC and BovEC infected with BTV-8. (J) Relative expression of IFIT1 (normalized to tubulin) in OvEC and BovEC. (K) Relative expression of Mx1 in OvEC and BovEC. For panels H to K, cells were pretreated with 4 μM Rux for 4 h prior to infection and maintained in the medium after infection. All data are from 3 independent experiments. Statistical significance was calculated using a two-way ANOVA performed using Tukey’s multiple-comparison test. *, *P* < 0.05; **, *P* < 0.01; ***, *P* < 0.001; ****, *P* < 0.0001.

Next, we investigated whether we could observe any species-specific differences in virus-induced host protein shutoff, a known mechanism used by BTV to modulate the cellular environment (65). To this end, we infected OvEC and BovEC with BTV-8 (using an MOI of 0.04 or 4) and then treated them with puromycin for 1 h at 4, 16, or 24 hpi prior to harvest. Incorporation of puromycin into nascent peptides provides an indication of cellular protein synthesis (66). As expected, BTV attenuated host protein synthesis both in OvEC and BovEC (Fig. 5A). However, when cells were infected with a high MOI, shutoff was essentially completed in OvEC by 16 hpi, while BovEC still supported nascent protein synthesis at 24 hpi (Fig. 5A and B). We next examined BTV protein expression, and NS1 was easily detectable in both OvEC and BovEC at 16 and 24 hpi (Fig. 5A). We found higher expression of NS1 in OvEC than BovEC at low MOI (Fig. 5C) (16 and 24 hpi, *P* < 0.05 by 2-way analysis of variance [ANOVA]), while at high MOI, the differences were not statistically significant (Fig. 5C). In addition, we observed that both pSTAT1 and STAT1 were downregulated, especially in OvEC (Fig. 5A and D).

During the IFN response, pSTAT1 is a key effector of the JAK/STAT signaling pathway leading to ISG upregulation. Both IFIT1 and Mx1 (as suitable ISG markers) were, as expected, upregulated in response to BTV infection (Fig. 5A, E, and F). However, when cells were infected at high MOI, downregulation of IFIT1 and Mx1 at late time points was apparent only in ovine cells. Downregulation of ISGs and STAT1 was most likely a result of generalized host protein shutoff, as opposed to specific targeting by the virus, as the expression patterns displayed by IFIT1, Mx1, and STAT1 were very similar to those observed by the housekeeping gene α-tubulin (Fig. 5A). As mentioned above, α-tubulin was used as a housekeeping gene control. Normally, α-tubulin is used as a gel loading control in each lane. However, in this series of experiments, because of virus-induced cellular shutoff, the intensity of the α-tubulin band was, as expected, significantly decreased in the samples where host protein synthesis was most downregulated. Hence, protein expression in each independent experiment was normalized to the average α-tubulin expression.

Similar experiments were also carried out in OvFib and BovFib (Fig. S3). Virus-induced protein shutoff in fibroblasts was slower in both sheep and cow fibroblasts than in infected EC, with no statistical difference of this process in these cell lines. This is also in line with BovFib possessing a reduced ability to hamper BTV replication compared to BovEC.

10.1128/mbio.00101-23.3 FIG S3 Virus-induced global host cell protein shutoff by BTV in ovine and bovine primary fibroblasts. (A) Immunoblotting of phosphorylated STAT1, total STAT1, Mx1, IFIT1, and BTV NS1 and protein translation rates (puromycin labeling treatment for 1 h) in OvFib and BovFib with BTV-8. Cells were infected at an MOI of 0.04 or 4 and harvested at the time points indicated. (B to F) Relative quantification of immunoblots, as shown in panel A, obtained from 3 independent experiments. (B) Quantification of relative protein shutoff over the course of BTV infection as determined by puromycin incorporation. All values were normalized to that of the mock-infected control (set to 1) for each time point. (C) Relative expression of BTV NS1 over time normalized to tubulin in OvFib and BovFib infected with BTV-8. (D) Expression of STAT1 (relative to the mock-infected control) over time normalized to tubulin in OvFib and BovFib infected with BTV-8. (E) Relative expression of IFIT1 over time normalized to tubulin in OvFib and BovFib. Cells were infected with BTV-8 using 2 different MOI and harvested at 3 different time points. Data are from 3 independent experiments. (F) Relative expression of Mx1 over time normalized to tubulin in OvFib and BovFib. Cells were infected with BTV-8 using 2 different MOI and harvested at 3 different time points. Data are from 3 independent experiments. Download FIG S3, TIF file, 2.7 MB. Copyright © 2023 Hardy et al. 2023 Hardy et al. https://creativecommons.org/licenses/by/4.0/ This content is distributed under the terms of the Creative Commons Attribution 4.0 International license.

We next assessed whether virus-induced protein shutoff is dependent on or independent of the virus-induced IFN response. We repeated the experiments described above, focusing on 24 hpi, as differences between cells from BTV-infected sheep and cattle were most evident at this time point. We performed infections in the presence of Rux or dimethyl sulfoxide (DMSO) (as a carrier control). Both Rux and DMSO were maintained in the medium for the duration of the experiment. Host protein shutoff was more pronounced in cells treated with Rux, in both OvEC and BovEC, than in those treated with DMSO (Fig. 5G). As expected, expression of BTV NS1 was increased in cells (especially BovEC) treated with Rux compared to DMSO-treated controls (Fig. 5H). Despite the Rux treatment, shutoff in BovEC was still not as pronounced as in OvEC, especially at low MOI (Fig. 5H). As expected, the treatment with Rux prevented STAT1 phosphorylation and upregulation of ISGs (Fig. 5G). As observed previously, in the presence of DMSO, the expression of Mx1 and IFIT1 was higher in BovEC infected with BTV-8 (Fig. 5J and K). These results indicate that the timing and extent of host protein shutoff are directly correlated with BTV replication levels. Blocking the IFN response by inhibiting the JAK/STAT pathway facilitates virus-induced host shutoff. On the other hand, protein shutoff at later time points negatively affects STAT1 phosphorylation and therefore the IFN response.

### Higher magnitude of the IFN response to BTV infection in bovine cells.

We next investigated whether we could identify in BTV-infected cells species-specific differences in the overall magnitude of the IFN response. We carried out transcriptomic analysis of endothelial cells infected at high MOI (~10) with either BTV-2 or BTV-8 (using 5 biological replicates from different donors) and focused on early stages of infection, at 6 and 12 hpi.

To gain an overall understanding of the cell transcriptomic reaction to BTV infection, we first used the Reactome pathway enrichment analysis (67, 68). At 6 hpi, in both bovine and ovine cells infected with either BTV-2 or BTV-8, the top 5 most significantly enriched pathways were all related to cytokine and interferon signaling and the immune system (Fig. S4). At 12 hpi, pathways unrelated to the immune response, such as metabolism of RNA, transcription, and others, were also significantly dysregulated in both bovine and ovine infected cells (Fig. S4). Interestingly, when we carried out the same analysis using only genes whose level of overexpression at 12 hpi was greater in infected bovine cells than sheep cells, we obtained only four significant overlapping enriched pathways: (i) interferon signaling, (ii) cytokine signaling in the immune system, (iii) IFN-α/β signaling, and (iv) immune system. Therefore, we next focused on analyzing the transcriptional regulation of the type I IFN system (genes expressing the type I IFN receptor, IFNAR1 and -2 genes, and ISGs) in BTV-infected cells.

10.1128/mbio.00101-23.4 FIG S4 Reactome pathway analysis of mapped differentially expressed genes in BTV-infected bovine and ovine endothelial cells. Pathways are indicated at 6 and 12 h postinfection for cells infected with either BTV-2 or BTV-8. The −log_10_(*Q* value) for the significant pathways identified are plotted. Download FIG S4, TIF file, 0.9 MB. Copyright © 2023 Hardy et al. 2023 Hardy et al. https://creativecommons.org/licenses/by/4.0/ This content is distributed under the terms of the Creative Commons Attribution 4.0 International license.

**FIG 6:**
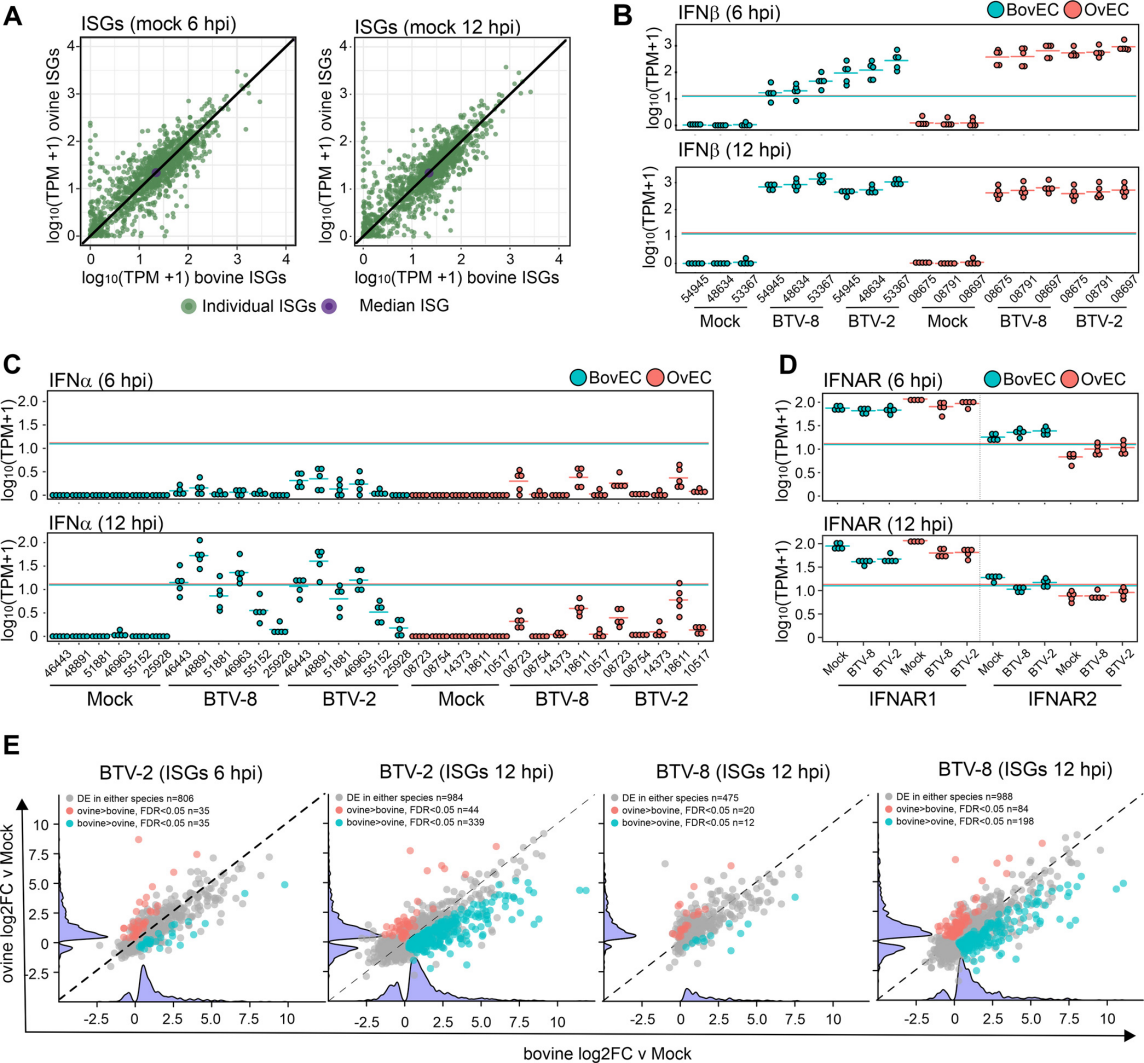
Transcriptomic response of BTV-infected OvEC and BovEC. (A) Scatterplot showing baseline regulation of ISGs in mock-infected OvEC and BovEC at 6 and 12 hpi as identified by RNA-seq. Values shown are transcripts per million [log_10_(TPM + 1)], and those identified in mock-infected OvEC are plotted against the log_10_(TPM + 1) in mock-infected BovEC. Individual ISGs are plotted in green, and the median values of the ISGs are plotted in purple. (B) Box-whisker plots of IFN-β transcripts in response to BTV-8 and BTV-2 infection in OvEC and BovEC at 6 and 12 hpi. (C) Box-whisker plots of IFN-α transcripts in response to BTV-8 and BTV-2 infection in OvEC and BovEC at 6 and12 hpi. (D) Box-whisker plots of IFNAR transcripts in response to BTV-8 and BTV-2 infection in OvEC and BovEC at 6 and 12 hpi. (E) Scatterplot showing differential expression (log_2_FC) of ISGs in OvEC and BovEC at 6 and 12 hpi in response to either BTV-2 or BTV-8 infection. Differentially expressed genes in both BovEC and OvEC are shown in gray, those only in OvEC are in red, and those only in BovEC are in blue. A violin plot of up- and downregulated genes for each species is represented on each axis.

In cultured cells, IFN-β is responsible for the first wave of the type I IFN response as a result of virus infection. Secreted IFN-β interacts with the type I IFN receptor, which induces a signaling cascade resulting in the induction of ISGs and a second wave of both IFN-β and IFN-α (69). Type I IFN genes in ruminants display numerous duplications and expansions, possessing at least 13 IFN-α and 6 IFN-β paralogues (70, 71). We concentrated only on orthologues displaying nonzero expression during virus infection. Importantly, we observed no baseline differences in ISG expression, suggesting that baseline IFN expression and downstream regulation are not different across species in the absence of infection in this model (Fig. 6A). In both BTV-2- and BTV-8-infected animals, there was a noticeable upregulation of IFN-β genes in infected sheep and cow cells, at both 6 and 12 hpi (Fig. 6B). At 6 hpi, there seemed to be a trend toward a higher upregulation of IFN-β genes in infected sheep cells, but there were no significant differences at 12 hpi. For IFN-α, levels of upregulation were minimal at 6 hpi, while at 12 hpi, we observed higher levels of upregulation in infected bovine cells (Fig. 6C). A similar pattern was observed with the anti-BTV ISGs IFIT1, RSAD2, and UBA7 (Fig. S5). In contrast, we observed no major differences between infected sheep and cow cells for the expression of type I IFN receptors (Fig. 6D).

10.1128/mbio.00101-23.5 FIG S5 Expression of ISGs with anti-BTV properties in infected bovine and ovine primary cells. Box-whisker plots of IFIT1, RSAD2, UBA7, OAS2 and HAS3 in response to BTV-8 and BTV-2 infection in OvEC and BovEC at 6 and 12 hpi. Download FIG S5, TIF file, 0.7 MB. Copyright © 2023 Hardy et al. 2023 Hardy et al. https://creativecommons.org/licenses/by/4.0/ This content is distributed under the terms of the Creative Commons Attribution 4.0 International license.

Next, we investigated whether we could identify any differences in the magnitude of the global ISG expression in infected sheep and cow cells. To model species-specific differences in ISG expression in cells infected with BTV-2 or BTV-8, we ran four separate generalized linear models (GLMs) (using edgeR’s glmQLFit function) (72, 73) (2 for BTV-2 and BTV-8 at both 6 and 12 hpi). We focused our analysis on ISGs that are present in both sheep and cattle, as we described previously (62). At 6 hpi, there were 801 and 473 ISGs that there were upregulated in BTV-2- and BTV-8-infected cells, respectively. Of these stimulated ISGs, only a relatively small number (~3 to 4%) were more highly upregulated in cells from one species than the other. At 12 hpi, approximately 900 ISGs were upregulated in both sheep and cow cells infected with either BTV-2 or BTV-8. However, in both cases there was a significant number of ISGs (26 to 40% of the total differentially expressed ISGs) that were more highly upregulated in infected cow cells than sheep cells. Importantly, these differences are limited to ISGs, as we noted no major differences when we carried out the same analyses with the whole transcriptome and excluded ISGs. Hence, these data confirmed that at the RNA level, BTV-infected cow cells display a more robust IFN response than infected sheep cells.

## DISCUSSION

Most animal reservoirs are tolerant to clinical disease, despite abundant virus replication that allows onward transmission to uninfected hosts (13). However, the mechanisms underpinning disease tolerance in reservoir species have not been investigated as widely as those occurring during disease pathogenesis in susceptible hosts. Understanding the complex virus-host interactions governing disease tolerance can allow us to interpret the critical balance between protective and damaging host responses (3). The host innate immune responses, and in particular type I IFN as one of its key effectors, plays a critical role in this balance, as it stimulates the expression of antiviral proteins, leading to host resistance, but at the same time can also exacerbate tissue lesions and disease severity through the release of proinflammatory factors (62, 74).

In this study, we used BTV, a virus of ruminants, as a model system to investigate the complex virus-host interactions correlating with disease susceptibility and tolerance. BTV infects a wide range of ruminants, resulting in drastically different clinical manifestations: while sheep in general develop clinical signs and can succumb to the disease, cattle remain mostly asymptomatic despite showing long-lasting viremia and being a reservoir of infection (25, 26, 38). We showed that the BTV replication kinetics are different in infections in cells derived from sheep and in cells from cattle. We used both primary skin fibroblasts and endothelial cells in our study, the latter being the key target of BTV replication and pathogenesis *in vivo* (37, 39). The phenotypes observed were similar in both cell types, although more pronounced in EC. We observed some variation between cell types and virus serotypes, but we accounted for the inherent variation of different primary cell preparations and the diverse genetic background by using distinct batches of cells from multiple donors and repeating the experiments independently several times. In addition, we used EC within 3 passages from isolation, with no freeze-thaw cycles between.

We determined that as early as 4 hpi, the replication kinetics of BTV were relatively hampered in cow cells compared to sheep cells. Hence, the very early events of BTV replication are more efficient in sheep cells.

Most importantly, we established that the differences in BTV replication kinetics in cow and sheep cells were largely abrogated by inhibiting the JAK/STAT pathway, whose activation leads to the upregulation of hundreds of ISGs (62). In addition, pretreatment with IFN had a relatively minor effect on the replication kinetics of BTV in sheep cells, while it substantially decreased viral replication in cow cells.

Hence, BTV shows a delayed replication kinetics in cow cells that is largely abrogated by the inhibition of the IFN response. Here, we identified several ISGs with anti-BTV properties: IFIT1, RSAD2, OAS2, UBA7, and HAS3. For IFIT1 and RSAD2, we were able to confirm their anti-BTV activity in both overexpression and gene knockout experiments. IFIT1 and RSAD2 have been shown before to possess antiviral activity for a wide variety of viruses. IFIT1 acts as a repressor of mRNA translation by interacting and interfering with the translation initiation factor eIF3 and acting on transcripts with 2′-O-methylation of the 5′ RNA cap structure (75). RSAD2 (viperin) catalyzes the conversion of CTP to 3′-deoxy-3′,4′-didehydro-CTP (ddhCTP), causing premature termination of RNA synthesis by the polymerase of various RNA viruses (76). However, we found no differences in the antiviral properties of the IFIT1 and RSAD2 orthologues or of any of the other anti-BTV ISGs identified in our study. These data therefore suggest that the effectors of both the sheep and cow IFN response are equally equipped to hamper the replication kinetics of BTV.

To overcome the innate immune response, many viruses, including BTV, employ host protein shutoff as a mechanism to suppress the translation of antiviral proteins (77–81). Indeed, we showed that protein shutoff was more pronounced and occurred at earlier time points after infection of sheep cells than cow cells. As expected, host protein shutoff included STAT1 (a phosphorylated protein essential for ISG expression) and antiviral ISGs such as MX1 and IFIT1, whose expression was lower in sheep cells than cow cells. Critically, host protein shutoff was not dependent on the IFN response, as it also occurred in the presence of an inhibitor of JAK/STAT signaling. On the contrary, host shutoff was more pronounced in cells treated with ruxolitinib, providing further evidence that this process is directly linked to the levels of BTV protein expression. Indeed, BTV-induced protein shutoff and modulation of the type I IFN response have been shown to be linked to expression of the viral proteins NS1, NS3, and NS4 (80–83).

Using RNA sequencing (RNA-seq) in BovEC and OvEC infected at a high MOI, we found no major differences in the induction of IFN-β or the type I IFN receptor (IFNAR1/2) at early time points postinfection. Indeed, ISG upregulation as a result of BTV infection was similar in sheep and cow cells at early time points after infection. However, at later time points (12 hpi), a higher number of ISGs were upregulated in cow than sheep cells. We also observed higher levels of upregulation of IFN-α at 12 hpi. Indeed, using biological assays, we found a more pronounced antiviral cytokine response in supernatants of bovine cells infected with BTV (at 24 hpi) than sheep cells. Interestingly, we noticed a similar trend also after exposure of the same cells to Sendai virus preparations containing defective interfering RNAs (Fig. S2). Hence, there is the possibility that bovine cells are intrinsically stronger producers of type I IFN than sheep cells, although this hypothesis needs to be tested with many different viruses and distinct cell types.

Our data collectively show that relatively small differences in viral replication at early time points determine a more efficient modulation of the type I IFN response in cells of tolerant hosts than cells of susceptible ones. The head start of BTV replication in sheep cells (evident as early as 4 hpi) results in a more abundant expression of viral proteins at earlier time points after infection, resulting in a faster virus-induced host protein synthesis shutoff compared to that in infected cow cells. The BTV NS1 protein has been shown to be a positive regulator of viral protein synthesis (80), and we showed it to be upregulated in sheep compared to cow cells as early as 6 hpi. Hence, BTV modulates the IFN response more effectively in sheep cells, as a direct result of virus-induced inhibition of translation of BTV restriction factors and any of the cellular proteins involved in the signaling pathways leading to ISG upregulation (as shown for STAT1). In addition, earlier expression of BTV proteins in infected sheep cells could also lead to an earlier modulation of the IFN response as a direct effect of the expression of IFN antagonists such as NS3 and NS4 (81–84). Conversely, in BTV-infected cow cells, a lower expression of viral proteins at earlier time points allows the IFN response to be activated at earlier time points and slow down viral replication. Importantly, the data obtained *in vitro* are compatible with previously published *in vivo* observations, showing a delayed onset of viremia in experimentally infected cattle compared to that in sheep (42).

Overall, our study suggests that the tipping point between tolerant and susceptible hosts during viral infection can likely be correlated with small differences in viral replication kinetics and the modulation of the IFN response. The innate immune response, and in particular the type I IFN response, are described as the first line of antiviral responses available to the host. However, in reservoir species, the balance between virus and innate immunity has been finely tuned through evolution to favor both host and virus survival. Counterintuitively, we propose a model where in reservoir species the timing and extent of the type I IFN response ultimately facilitates virus transmission from infected to uninfected hosts.

It will be extremely important to determine the mechanisms underpinning disease tolerance not only to understand important lessons in virus emergence and pathogenesis but also to provide new intellectual frameworks for the development of host-directed antiviral therapeutic strategies (6).

## MATERIALS AND METHODS

### Cells.

BSR (a clone of BHK21, kindly provided by Karl Conzelmann) and CPT-Tert cells were maintained as previously described (82, 85). *Ex vivo* samples from healthy cattle and sheep (ears and aortas) were harvested from abattoirs and used to isolate primary fibroblasts and endothelial cells following previously published protocols (62, 86). All isolated cells were tested for pestiviruses and mycoplasma prior to use, and passages of the primary cells were kept to a minimum. We took care to use multiple donors to prepare primary cells.

Bovine and ovine primary cells were also immortalized by transducing cells with a retrovirus expressing the catalytic subunit of human telomerase (hTERT), as previously described (87). Briefly, cells were immortalized using the catalytic subunit of hTERT delivered by transduction using a lentivirus vector (pLV-hTERT-IRES-Hygro; Addgene). Two days postransduction, cells were selected with 200 μg/mL of hygromycin for 7 days and maintained as a bulk population for fibroblasts. Immortalized cell lines generated in this study are referred to a BovEChTert, OvEChTert, BovFibT, and OvFibT. Morphology, growth rate, and IFN responses of immortalized fibroblasts and endothelial cells were monitored over time (up to passage 15) and were similar to those of corresponding bovine and ovine primary cells. As previously described, this system has been demonstrated to extend the life span of the cells without compromising interferon signaling (87, 88). Single-cell clones of endothelial cells were selected, amplified, and screened for interferon competency.

In some experiments, as indicated below, cells were treated with Rux (APEXBio; a potent inhibitor of the JAK/STAT pathway), uIFN (PBL InterferonSource), and puromycin dihydrochloride (Sigma-Aldrich).

### Viruses.

Two serotypes of BTV were used in this study, BTV-8 and BTV-2, as previously described (32, 82). BTV-8-mGFP, expressing a monomeric GFP in the C terminus of the NS1 open reading frame, was generated by reverse genetics using established methods (82, 89, 90). Upon rescue, BTV-8-mGFP was plaque purified for the generation of working stocks. BTV-8-mGFP maintained GFP expression for at least two passages, and aliquots of passage 2 were stored at −70°C and were used to generate the working stock used in all the experiments.

The replication kinetics of BTV were determined in ovine and bovine primary cells. Cell monolayers in a 12-well plate were infected at an MOI of 0.01 (as assessed in CPT-Tert) under different conditions. To inhibit the JAK/STAT pathway, cells were pretreated for 4 h with 4 μM Rux or mock treated with the equivalent volume of DMSO diluent. Cells were then maintained in medium supplemented with Rux for the duration of the experiments. To stimulate an antiviral state, cells were pretreated with 500 to 1,000 IU/mL of uIFN for 24 h prior to infection, and uIFN was used to supplement the medium for the duration of the experiment.

At different time points postinfection, 100 μL of supernatant was collected from each well and replaced with 100 μL of the appropriate cell medium. Supernatants were stored at 4°C until they were processed for titration. Virus titers were determined by endpoint dilution analysis on BSR cells and expressed as log_10_ 50% tissue culture infective doses (TCID_50_)/mL (91, 92). Each virus growth curve experiment was performed at least 3 times independently, and each experiment was carried out in triplicate. At least two independently generated virus stocks were used for each virus.

Preparations of Sendai virus (SeV; Cantell strain) were purchased from Charles River Laboratories. Virus-like particles (VLPs) of VSV with the envelope protein deleted but decorated with the VSV G protein in *trans* during VLP production (VSV-ΔG-GFP) were used in biological assays to assess the antiviral state of primary cells infected with either BTV or SeV as described previously (93, 94). Briefly, BovEC, OvEc, BovFib, and OvFib were cultured in 12-well plates and infected with either BTV-8 or SeV at MOI of 1 for 24 h. Supernatants were then collected, UV inactivated, and stored at −80°C. Antiviral cytokines produced by the primary cells were quantified in indicator cells (CPT-Tert) stimulated for 24 h with serial dilutions of UV-inactivated supernatants or uIFN as a standard control. Stimulated cells were washed and infected with VSV-ΔG-GFP for 6 h before being fixed in 4% formaldehyde. Cells were then analyzed by flow cytometry (Guava easyCyte; Millipore). The levels of antiviral cytokines produced by BTV- and SeV-infected cells were estimated by comparing the levels of protection to VSV-ΔG-GFP induced by supernatants of infected cells to those by known amounts of uIFN. Similar assays were carried out in BovEC, OvEc, BovFib, and OvFib stimulated with serial dilutions of uIFN before challenge with VSV-ΔG-GFP.

### Visualization of cytopathic assay in primary cells.

Primary ovine and bovine endothelial cells were infected in 12-well plates as described previously. Infected cells were incubated at 37°C and fixed in 4% (wt/vol) formaldehyde in phosphate-buffered saline (PBS) (FS) at 24, 48, 72, and 96 hpi. Monolayers were stained with Coomassie blue staining solution (0.1% [wt/vol] Coomassie brilliant blue R-250, 45% [vol/vol] methanol, 10% [vol/vol] glacial acetic acid) and imaged with a photo scanner (Epson Expression 1680 Pro).

### Bovine ISG library.

A bovine arrayed ISG library was synthesized (Genewiz) in the pSCRPSY lentiviral vector, allowing the coexpression of the gene of interest and a fluorescent protein (TagRFP), as previously described for human and macaque ISG libraries (58, 63). In total, 289 unique bovine genes and 25 additional cDNA sequences accounting for splicing variants were included in the library (Table S1). They correspond to the repertoire of the most positively upregulated genes identified in bovine primary cells following IFN treatment (62) in addition to the mammalian core ISGs evolutionarily conserved across mammalian and chicken genomes. Lentivirus vectors expressing the ISG library were generated in the 96-well plate format as described previously (58), with one ISG per well, by cotransfecting 3.5 × 10^4^ adherent 293T cells with 125 ng of the ISG construct in the SCRPSY vector and plasmids expressing HIV Gag-Pol (NLGP) and VSV-G in a ratio of 25:5:1. The medium was changed 24 h posttransfection, and supernatants were subsequently harvested at 48, 72, and 96 h and stored at −80°C. Independent stocks of lentiviruses were generated for validation assays.

### Bovine ISG screens.

293T cell suspensions were transduced via spinoculation for 1 h at 500 × *g* at 4°C in the presence of 8 μg/mL Polybrene. The percentage of RFP-positive cells is indicative of the transduction efficiency and was used as an indicator of ISG expression levels. Forty-eight hours postransduction, the cell populations were split and infected with 50 μL of BTV-8-mGFP virus for a period of 16 h or 32 h. The dose of BTV-8-mGFP had previously been optimized to ensure that 10 to 50% of cells in each well were infected. For the late screen, a final concentration of 0.625 μg/mL of puromycin was used to supplement the medium, enabling virus replication to be limited to the transduced cells. At 16 and 32 hpi, cells were washed, trypsinized, and fixed in 4% (wt/vol) formaldehyde in PBS. Cells were then analyzed by flow cytometry, using the Guava easyCyte instrument (Millipore) equipped with a 488-nm and a 532-nm laser. For each well, the single live cell population was gated and levels of GFP and RFP were measured. ISGs with transduction levels (measured by RFP expression) below 10% were excluded from downstream analyses. Data were then analyzed using FlowJo (TreeStar) software.

### Western blotting.

Cells were lysed as described previously (95). Samples were subjected to SDS-PAGE, and immunoblotting was performed using the following antibodies: puromycin (Millipore; MABE343), pSTAT1 (Cell Signaling; 9167S), STAT1 (Santa Cruz; sc-592), IFIT1 (Origene; TA500948), BTV NS1 (82), RSAD2 (Proteintech; 11833-1-AP), α-tubulin (Proteintech; 66031-1-Ig), and glyceraldehyde-3-phosphate dehydrogenase (GAPDH) (Cell Signaling; 2118S). Mx1 antibody was kindly provided by Georg Kochs (University Medical Centre, Freiburg, Germany). The secondary antibodies used for immunoblotting were anti-rabbit IgG (H+L) (DyLight 800 4X polyethylene glycol [PEG] conjugate) (Cell Signaling; 5151S) and anti-mouse IgG (H+L) (DyLight 680 conjugate) (Cell Signaling; 5470S). Proteins were visualized using the Odyssey CLx imager, and quantification was performed using Image Studio Lite software (LI-COR Biosciences).

### CRISPR/Cas9 knockout cells.

Target-specific single guide RNAs (sgRNAs) for bovine IFIT1 and RSAD2 (*IFIT1* forward, 5′-caccgAATCGTTGTCTATCGCCTGG-3′; *IFIT1* reverse, 5′-aaacCCAGGCGATAGACAACGATTc-3′; *RSAD2* forward, 5′-caccgAGTGGTAATTGACGCTGGTG-3′; *RSAD2* reverse, 5′-aaacCACCAGCGTCAATTACCACTc-3′; in lowercase sequences used for cloning purposes) were designed using the CHOPCHOP web tool (https://chopchop.cbu.uib.no/) and cloned into pLentiCRISPR v2 vector (Addgene) as described previously (96, 97). Lentivirus were made as described above and used to transduce immortalized bovine cells (BovEChTert and BovFibhTert). Cells were selected in the presence of 2.5 mg/mL puromycin and single-cell population expanded. To ensure that resulting cells maintained their responsiveness to interferon and that the gene of interest had been knocked out, cells were treated with 500 to 1,000 U uIFN for 24 h at 37°C and processed for immunoblotting for relevant proteins. For each ISG, two clones with the greatest reduction in specific protein expression following uIFN treatment were defined as KO bovine fibroblasts and used for further experimentation.

### BTV-mGFP infectivity in primary bovine and ovine endothelial cells.

BovEC and OvEC from aortas collected from three different animals were seeded in 96-well plates. Twofold serial dilutions of BTV-8-mGFP were used to spinoculate cells at 500 × *g* for 1 h at 4°C. The inoculum was then removed, and cells were washed with serum-free medium once before the addition of warm endothelial cell growth medium. Infected cells were incubated at 37°C for 6 h and then fixed in 4% (wt/vol) FS. The proportion of GFP-expressing cells was then determined by flow cytometry (Guava easyCyte; Luminex) by acquiring at least 10,000 events. In addition, OvEC, BovEC, and immortalized OvEChTert and BovEChTert were infected with BTV-mGFP (MOI of either 0.3 or 1) and fixed at either 2, 4, or 6 hpi. The percentage of GFP-expressing cells and the mean fluorescence intensity (MFI) were obtained by flow cytometry.

### Binding assays.

OvEChTert and BovEChTert cells were seeded in a 12-well plate and at confluence were infected at an MOI of 10 synchronously at 4°C by spinoculation as described above. The inoculum was aspirated, and cells were washed in ice with cold medium twice to remove unbound virus. Aliquots of the wash were kept for RNA extraction. Medium (prewarmed to 37°C) was added directly to the cells, which were further incubated at 37°C. At the specified time postinfection, medium was collected, and TRIzol (Invitrogen) was directly added to the monolayer. RNA was extracted from the samples as described before (62), and the number of BTV viral genome equivalents was determined by quantitative RT-PCR based on the number of copies of BTV segment 10 as described before (98). All reactions were performed on ABI 7500 Fast thermal cycler.

### Protein shutoff assays.

To examine protein shutoff, nascent proteins were labeled with puromycin as previously described (86). Primary ovine and bovine cells were infected with BTV-8 at an MOI of 0.04 or 4 by spinoculation as described above. After 4, 16, and 24 hpi, puromycin was added to the cells at a final concentration of 3.3 ng/mL for 1 h. Following cell lysis, samples were subjected to SDS-PAGE, and immunoblotting was performed for puromycin and various other proteins as indicated. For experiments in the presence of Rux, cells were pretreated with 4 μM Rux for 4 h prior to spinoculation at 500 × *g*, and Rux-supplemented medium (or the DMSO control) was added back to the cells for the duration of the experiment. At 24 hpi, 3.3 ng/mL puromycin was added to the cells for 1 h. Cells were then lysed and processed for immunoblotting as mentioned above.

Relative quantification of puromycin, Mx1, IFIT1, STAT1, and NS1 was performed by normalizing against average α-tubulin for each condition to compensate for the reduction in expression of the housekeeping genes due to protein shutoff. For the quantification of the puromycin labeling in infected samples, the value of each sample was normalized to that of mock-infected cells (set to 1). Relative quantification of other viral and cellular proteins mentioned above used the highest value (set to 1) for each MOI in each experiment.

### RNA-seq.

Primary EC were infected in 12-well plates at an MOI of ~5 with either BTV-8 or BTV-2 by spinoculation at 500 × *g* at 4°C for 1 h. Cells were then further incubated 1 h at 4°C. Medium was then removed, and cells were washed with warm medium before incubation at 37°C for either 6 or 12 hpi. Monolayers were then washed with warm PBS to remove any cellular debris and immediately lysed in 750 μL of TRIzol. Total RNA was extracted from each sample as previously published (62). Sample RNA concentration was measured with a Qubit fluorimeter (Life Technologies), and the RNA integrity was determined using an Agilent 4200 TapeStation. Samples had an average RNA integrity number of ~9.8, and 500 ng of total RNA from each sample was taken for library preparation. Libraries for sequencing were generated using an Illumina TruSeq stranded-mRNA HT kit, according to the manufacturer’s instructions. Briefly, polyadenylated RNA molecules were captured, followed by fragmentation. RNA fragments were reverse transcribed and converted to dsDNA, end repaired, A tailed, ligated to indexed adaptors, and PCR amplified. Libraries were pooled in equimolar concentrations and sequenced in Illumina NextSeq 500 and 550 sequencers using a high-output cartridge, generating single reads with a length of 75 bp. At least 94.77% of the reads generated presented a quality score of 30 or above.

RNA-seq read quality was assessed using the FastQC software, and sequence adaptors were removed using TrimGalore. The reference Ovis aries genome (Oar_v3.1) and the Bos taurus genome (ARS-UCD1.2) were downloaded from the Ensembl genome browser, and RNA-seq reads were then aligned to their respective host genomes. Following the alignment, FeatureCount (99) was used to count reads mapping to genes in annotation files. The edgeR package was then used to calculate the gene expression levels and to analyze differentially expressed genes (73).

To model the species-specific responses to infection with two different BTV strains, four separate GLMs were run using edgeR’s glmQLFit function: 2 for BTV-2 and BTV-8 at both 6 and 12 h postinfection. For each GLM, the five replicate read counts from each of the four conditions (bovine, ovine, infected, and uninfected) were used, giving 5 × 4 sets of counts as input for each GLM. These counts were used to generate four effect terms. Each effect term describes the log_2_ fold change (log_2_FC) between the conditions, with an associated false discovery rate (FDR) for each term to test for statistical significance of the effect: (i) an uninfected sheep cell term, describing baseline expression in uninfected cells, and a term for cow expression relative to sheep; (ii) a cow term providing the cow-specific baseline expression in the cell lines; (iii) an infection term describing the log_2_FC between uninfected and infected sheep; and (iv) a cow-by-infection interaction term describing the difference in the way the two species’ cell lines respond to infection. Partitioning these effects allowed the species-specific response to infection to be determined, thus avoiding the confounding effects of baseline expression that would be involved in a simple pairwise comparison across species. The fact that each effect is associated with an FDR contextualizes the effect size.

A model matrix for each GLM was also calculated. An intercept was calculated to provide the baseline value for bulk uninfected sheep cell sequencing. Whether ovine or bovine data provided the baseline intercept was arbitrary; this simply inverted the second and fourth species-specific effect terms. Transcripts per million were calculated as follows: $\frac{X_{i}}{l_{i}}\times 10^{6}÷\sum \frac{X_{i}}{l_{i}}$, where *X_i_* represents the count number of reads mapping to each gene and *l_i_* represents the length of each gene. To gain an overall understanding of the cell transcriptomic reaction to BTV infection, we used the Reactome pathway enrichment analysis suite on each species’ response to infection (67, 68, 100). We mapped ovine Ensembl IDs to human Entrez IDs for this package using the biomaRt package in R (101).

### Statistical analyses.

Statistical analyses were performed using the GraphPad Prism software. Quantitative data were presented as arithmetic means and standard errors of the means (SEM). Statistical significance of differences in puromycin labeling between mock infections and infections at MOI of 0.04 or 4 at different time points was assessed using unpaired *t* tests with the Bonferroni posttest to compare samples at each MOI with the mock-infected sample. The same method was used to assess differences in NS1, Mx1, IFIT1, and RSAD2 expression between ovine and bovine samples at different time points. Differences in means were considered significant if the *P* value was <0.05.

### Data availability.

The raw FASTQ files generated during this project have been submitted to the European Nucleotide Archive (ENA) under project accession number PRJEB55791.
