# Supplementary figures and images for: The Timing and Magnitude of the Type I Interferon Response Are Correlated with Disease Tolerance in Arbovirus Infection
Source: mBio. 2023 Apr 25;14(3):e00101-23. doi: 10.1128/mbio.00101-23 (PMC10294695; doi:10.1128/mbio.00101-23)

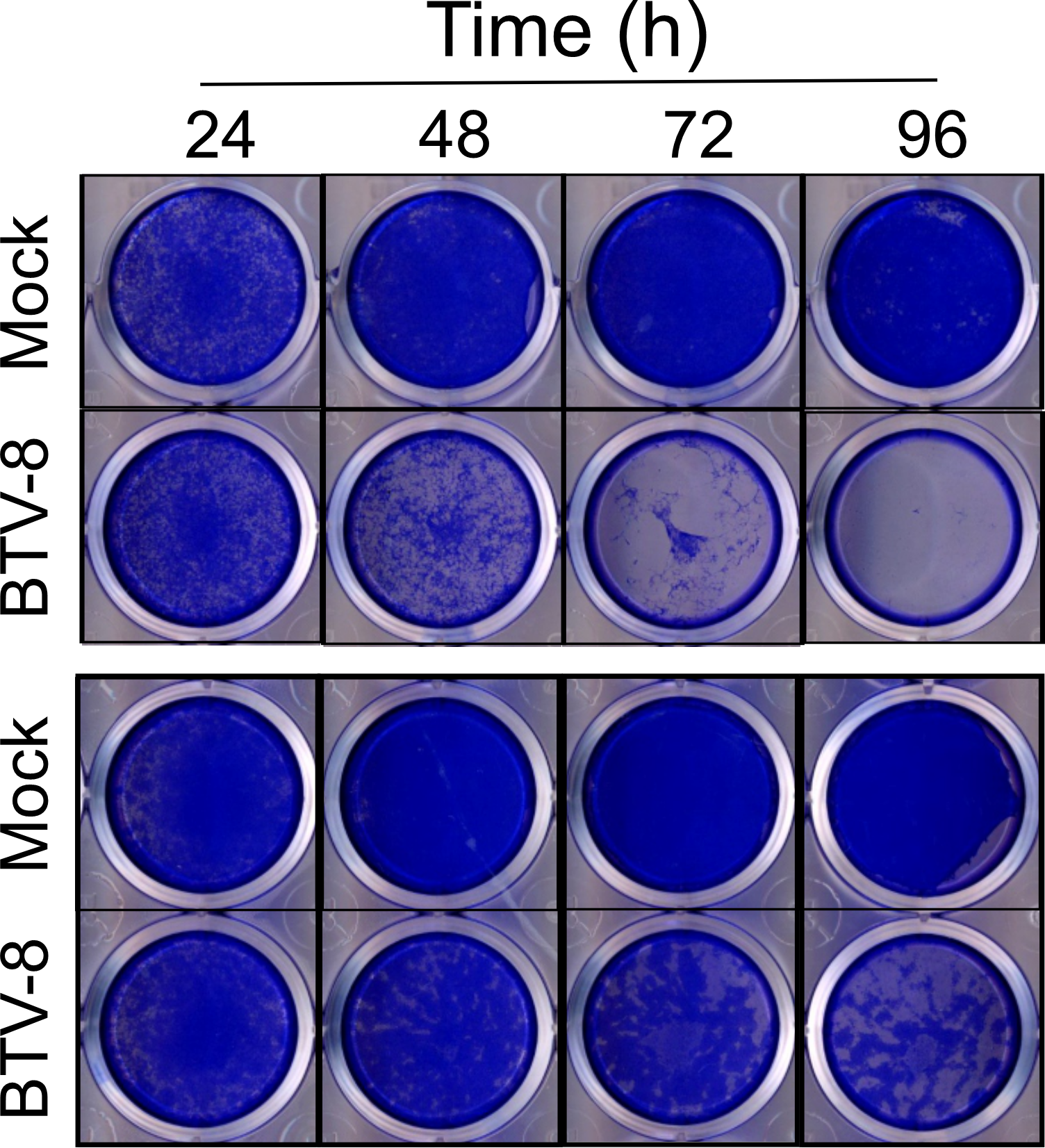

Supplement: FIG S1 [file mbio.00101-23-s0001.tif]

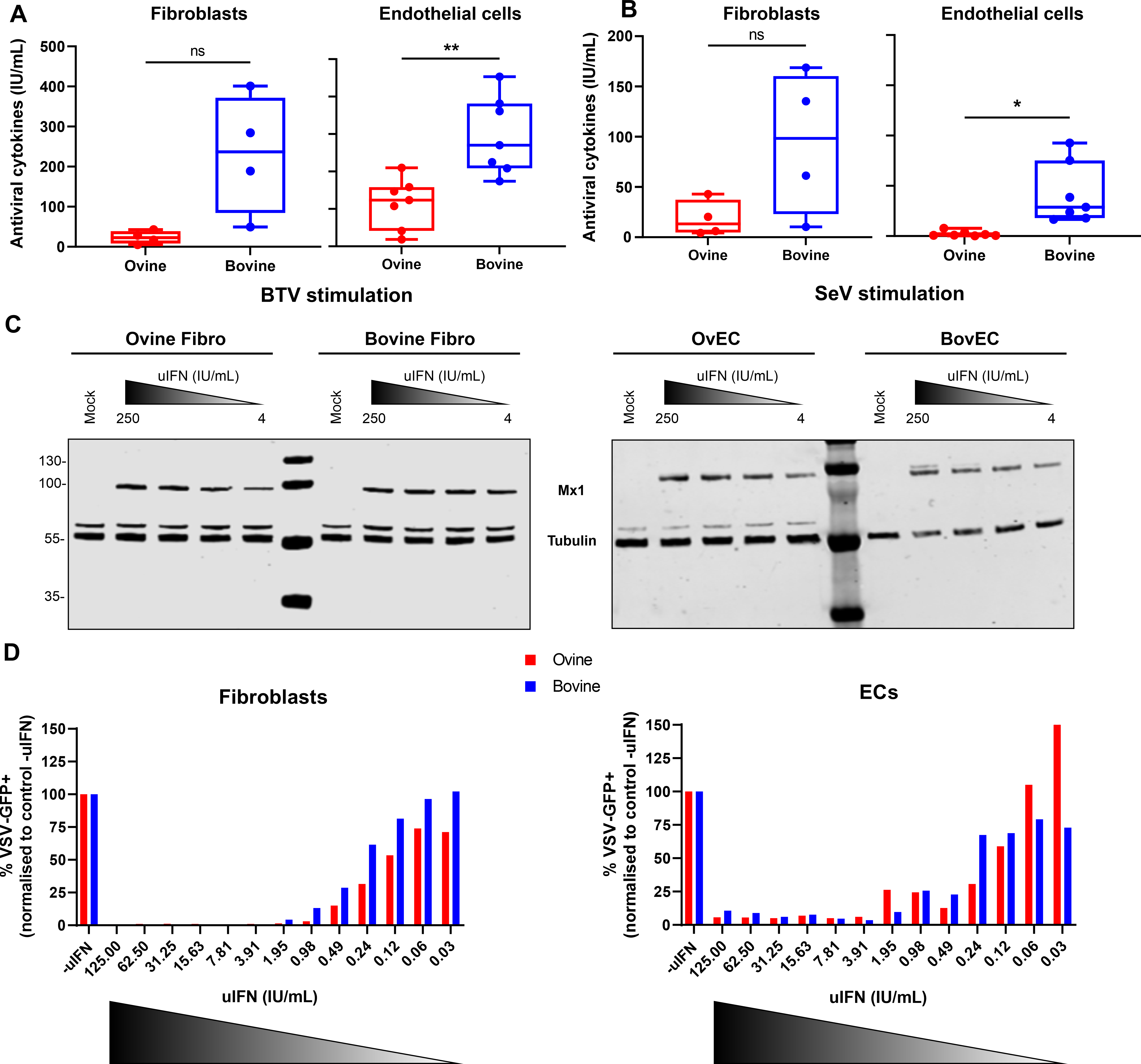

Supplement: FIG S2 [file mbio.00101-23-s0002.tif]

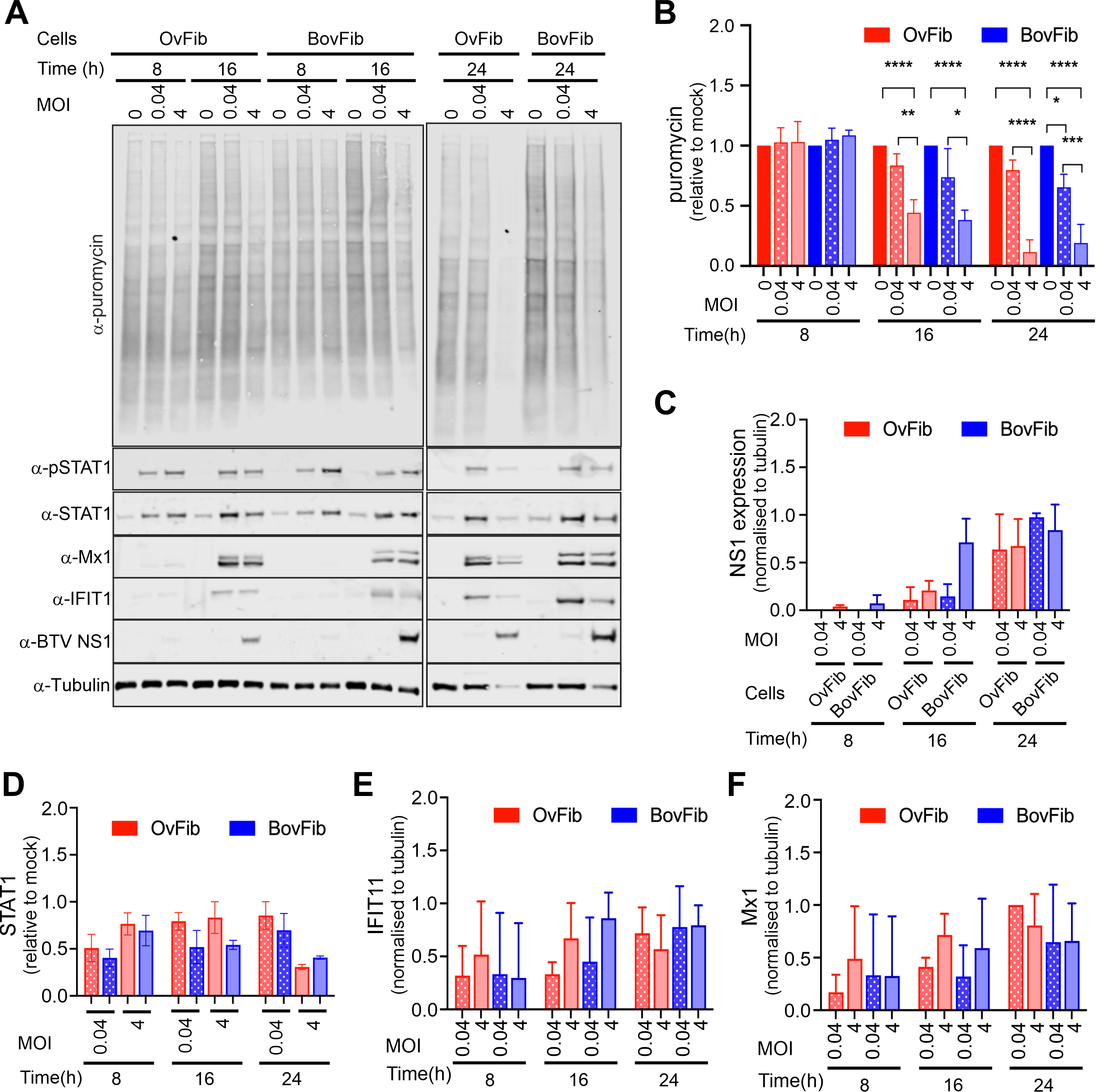

Supplement: FIG S3 [file mbio.00101-23-s0003.tif]

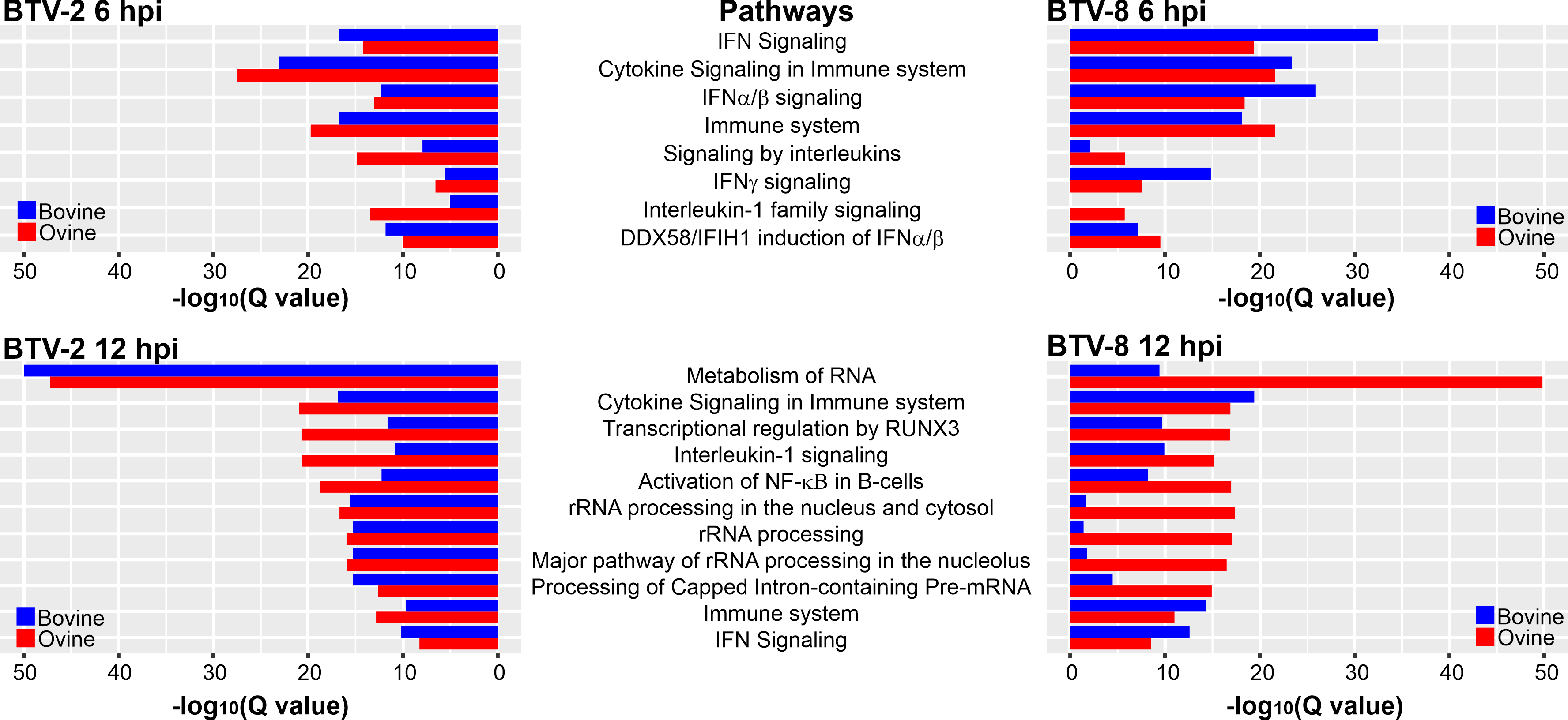

Supplement: FIG S4 [file mbio.00101-23-s0004.tif]

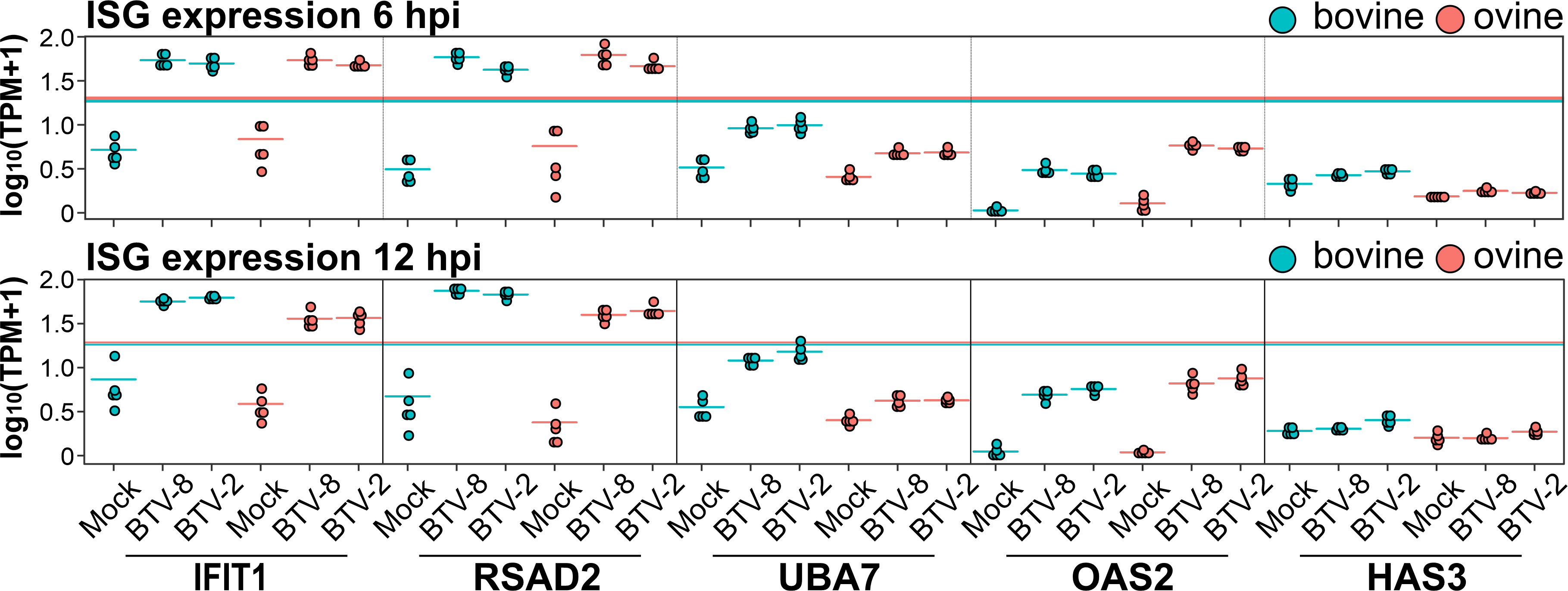

Supplement: FIG S5 [file mbio.00101-23-s0005.tif]
